# Growth in stratospheric chlorine from short‐lived chemicals not controlled by the Montreal Protocol

**DOI:** 10.1002/2015GL063783

**Published:** 2015-06-01

**Authors:** R. Hossaini, M. P. Chipperfield, A. Saiz‐Lopez, J. J. Harrison, R. von Glasow, R. Sommariva, E. Atlas, M. Navarro, S. A. Montzka, W. Feng, S. Dhomse, C. Harth, J. Mühle, C. Lunder, S. O'Doherty, D. Young, S. Reimann, M. K. Vollmer, P. B. Krummel, P. F. Bernath

**Affiliations:** ^1^ School of Earth and EnvironmentUniversity of LeedsLeedsUK; ^2^ Atmospheric Chemistry and Climate GroupInstitute of Physical Chemistry Rocasolano, CSICMadridSpain; ^3^ National Centre for Earth Observation, Department of Physics and AstronomyUniversity of LeicesterLeicesterUK; ^4^ Centre for Ocean and Atmospheric Sciences, School of Environmental SciencesUniversity of East AngliaNorwichUK; ^5^ Now at Department of ChemistryUniversity of LeicesterLeicesterUK; ^6^ Rosenstiel School of Marine and Atmospheric ScienceUniversity of MiamiMiamiFloridaUSA; ^7^ National Ocean and Atmospheric AdministrationBoulderColoradoUSA; ^8^ National Centre for Atmospheric ScienceUniversity of LeedsLeedsUK; ^9^ Scripps Institution of OceanographyUniversity of CaliforniaSan DiegoCaliforniaUSA; ^10^ Monitoring and Information Technology DepartmentNorwegian Institute for Air ResearchKjellerNorway; ^11^ Atmospheric Chemistry Research Group, School of ChemistryUniversity of BristolBristolUK; ^12^ Swiss Federal Laboratories for Materials Science and TechnologyDübendorfSwitzerland; ^13^ Oceans and Atmosphere FlagshipCSIRO AspendaleVictoriaAustralia; ^14^ Department of Chemistry and BiochemistryOld Dominion UniversityNorfolkVirginiaUSA

**Keywords:** dichloromethane, VSLS, ozone, stratosphere, Montreal Protocol, phosgene

## Abstract

We have developed a chemical mechanism describing the tropospheric degradation of chlorine containing very short‐lived substances (VSLS). The scheme was included in a global atmospheric model and used to quantify the stratospheric injection of chlorine from anthropogenic VSLS ( ${\text{Cl}}_{y}^{\text{VSLS}}$) between 2005 and 2013. By constraining the model with surface measurements of chloroform (CHCl_3_), dichloromethane (CH_2_Cl_2_), tetrachloroethene (C_2_Cl_4_), trichloroethene (C_2_HCl_3_), and 1,2‐dichloroethane (CH_2_ClCH_2_Cl), we infer a 2013 ${\text{Cl}}_{y}^{\text{VSLS}}$ mixing ratio of 123 parts per trillion (ppt). Stratospheric injection of source gases dominates this supply, accounting for ∼83% of the total. The remainder comes from VSLS‐derived organic products, phosgene (COCl_2_, 7%) and formyl chloride (CHClO, 2%), and also hydrogen chloride (HCl, 8%). Stratospheric ${\text{Cl}}_{y}^{\text{VSLS}}$ increased by ∼52% between 2005 and 2013, with a mean growth rate of 3.7 ppt Cl/yr. This increase is due to recent and ongoing growth in anthropogenic CH_2_Cl_2_—the most abundant chlorinated VSLS not controlled by the Montreal Protocol.

## Key Points

- Stratospheric Cl from short‐lived chemicals has increased significantly
- Increasing Cl due to rapid growth in surface emissions of CH_2_Cl_2_
- COCl_2_ and HCl from VSLS make a nonzero contribution to stratospheric Cl

## 1 Introduction

In addition to long‐lived source gases, such as chlorofluorocarbons and halons, halogenated very short‐lived substances (VSLS)—with lifetimes in the lower troposphere <6 months—are a source of stratospheric chlorine and bromine [e.g., *Sturges et al.*, 2000; *Mébarki et al.*, 2010]. VSLS enhance ozone (O_3_) loss rates in the lower stratosphere [e.g., *Salawitch et al.*, 2005], where O_3_ perturbations exert a relatively large impact on climate [*Riese et al.*, 2012; *Saiz‐Lopez et al.*, 2012; *Hossaini et al.*, 2015]. Quantification of stratospheric VSLS loading has, therefore, been the objective of many recent field measurements and modeling studies [e.g., *Hossaini et al.*, 2012; *Tegtmeier et al.*, 2013; *Kreycy et al.*, 2013; *Sala et al.*, 2014; *Liang et al.*, 2014; *Fernandez et al.*, 2014]. In particular, constraining oceanic emissions and the troposphere‐to‐stratosphere transport of natural brominated VSLS has been at the forefront, as these VSLS account for a significant portion (∼25%) of total stratospheric bromine. Chlorinated VSLS contribute a smaller relative contribution to total stratospheric chlorine (at present a few percent) but are mostly of anthropogenic origin [e.g., *Simmonds et al.*, 2006] and are not controlled by the Montreal Protocol.

The most abundant chlorinated VSLS are CH_2_Cl_2_ and CHCl_3_ for which anthropogenic activity accounts for ∼90% and ∼25% of their tropospheric abundance, respectively [*Montzka et al.*, 2011]. CHCl_3_ is used in the manufacture of certain hydrofluorocarbons (HFCs) and is a by‐product of water chlorination and bleaching processes. CH_2_Cl_2_ is a solvent used for paint removal, foam production, and as a feedstock for HFC production [*Campbell et al.*, 2005]. Source gas injection (SGI) of these VSLS, together with relatively minor species such asC_2_Cl_4_ and C_2_HCl_3_, is estimated to provide 72 (50–95) parts per trillion (ppt) Cl to the stratosphere [*Carpenter et al.*, 2014]. This estimate, based mostly on aircraft VSLS observations, is appropriate for 2012 and does not reflect the rapid growth in surface CH_2_Cl_2_ observed over the 2012–2013 period [*Hossaini et al.*, 2015].

In addition to SGI, it is hypothesized that VSLS‐derived phosgene (COCl_2_) and hydrogen chloride (HCl), produced in the troposphere, also reach the stratosphere. Based on measured COCl_2_ and HCl in the tropical tropopause layer (TTL), the estimated stratospheric product gas injection (PGI) of these gases contributes 25 (0–50) ppt Cl [*Carpenter et al.*, 2014]. However, observations alone cannot distinguish VSLS‐derived products from those derived from other sources and this is reflected in the large uncertainty range given on the above PGI estimate. This uncertainty has been acknowledged for some time in Ozone Assessment Reports [e.g., *Law et al.*, 2007; *Montzka et al.*, 2011; *Carpenter et al.*, 2014], though modeling work to resolve the issue has yet to be performed.

In this study, we developed a chemical degradation mechanism for chlorinated VSLS. The scheme was included in a global model, and simulations were performed to (1) quantify stratospheric ${\text{Cl}}_{y}^{\text{VSLS}}$, (2) assess the relative contribution of SGI versus PGI, and (3) examine the trend in stratospheric ${\text{Cl}}_{y}^{\text{VSLS}}$ over the 2005–2013 period.

## 2 Model and Experiments

TOMCAT is an off‐line three‐dimensional chemical transport model [*Chipperfield*, 2006]. The model has been widely used for studies of tropospheric composition, including previously the chemistry and transport of brominated VSLS [e.g., *Hossaini et al.*, 2013, 2010, 2012]. TOMCAT is forced by meteorological parameters, including wind and temperature fields, taken from the European Centre for Medium‐Range Weather Forecasts ERA‐Interim reanalyses [*Dee et al.*, 2011]. The model contains parameterizations of mixing in the boundary layer [*Holtslag and Boville*, 1993] and moist convection [*Tiedtke*, 1989]. Large‐scale vertical transport is calculated through divergence of the horizontal winds, and tracer advection follows the scheme of *Prather* [1986]. The model was run at a horizontal resolution of 2.8°×2.8° and with 31 vertical (*σ*‐*p*) levels from the surface to ∼30 km.

The version of TOMCAT used here contains a comprehensive tropospheric chemistry scheme including O_*x*_‐NO_*y*_‐HO_*x*_‐C_1_‐C_3_ nonmethane hydrocarbons, isoprene, and bromine chemistry [*Breider et al.*, 2010]. A gas‐phase chlorine chemistry scheme has been added to the model along with five chlorinated VSLS tracers; CHCl_3_, CH_2_Cl_2_, C_2_Cl_4_, C_2_HCl_3_, and CH_2_ClCH_2_Cl. Loss of these VSLS, primarily through OH‐initiated oxidation, yields a range of organic products, including phosgene, COCl_2_, based on the general halocarbon degradation mechanism outlined in *Ko et al.* [2003]. A description of tracers and the model chemistry scheme is given in the supporting information.

In all simulations, background tropospheric chlorine was supplied from the breakdown of (i) ocean‐emitted halocarbons (e.g., CHBr_2_Cl, CH_2_BrCl, and CHBrCl_2_), whose emissions were specified [*Ordóñez et al.*, 2012], and (ii) the relatively long‐lived source gas methyl chloride (CH_3_Cl). The stratospheric‐relevant source gases carbon tetrachloride (CCl_4_) and methyl chloroform (CH_3_CCl_3_), not routinely included in this tropospheric model configuration, were also considered as they, in addition to VSLS, are a COCl_2_ source.

Three simulations covering the 2005–2013 period were performed. EXP1 (control run) contained no anthropogenic chlorinated VSLS (only natural Cl sources). EXP2 was identical to EXP1 but also contained CHCl_3_, CH_2_Cl_2_, and C_2_Cl_4_. A mixing ratio boundary condition, as opposed to specified emissions, was used to constrain their surface abundance in the model. This varied with latitude (five bands, >60°N, 30–60°N, 0–30°N, 0–30°S, and >30°S) and annually, based on surface observations from the NOAA and AGAGE monitoring networks [e.g., *Prinn et al.*, 2000; *Montzka et al.*, 2011]; see supporting information. With this boundary condition, the model was then used to simulate the tropospheric distribution and troposphere‐to‐stratosphere transport of VSLS and their product gases. EXP3 was identical to EXP2 but also included C_2_HCl_3_ and CH_2_ClCH_2_Cl. Long‐term surface observations of these VSLS are sparse; therefore, they were only “switched on” in the model post 2010 and their surface abundance was scaled to give reasonable agreement with observed upper tropospheric mixing ratios (see section 3.1).

## 3 Results and Discussion

### 3.1 Source Gas Injection

**Figure 1:**
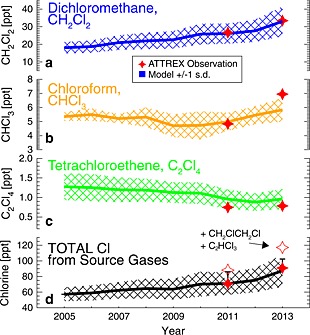
Time series of simulated tropical mean (a) CH_2_Cl_2_, (b) CHCl_3_, and (c) C_2_Cl_4_ mixing ratio (ppt) at the level of zero radiative heating (LZRH) (grated area, ±1 standard deviation) derived from observed surface values. Equivalent observed quantities at the LZRH from the 2011 (114° to 134°W longitude and 7° to ∼20°N latitude, November) and 2013 (92° to 172°W longitude and 0.2° to ∼20°N latitude, February/March) NASA ATTREX missions are also shown. (d) The summed total chlorine in source gases; 3 × CHCl_3_+2 × CH_2_Cl_2_+4 × C_2_Cl_4_, from model run EXP2 and observed quantity shown with filled stars. The 2011 and 2013 error bars correspond to the total chlorine in source gases when 3 × C_2_HCl_3_+2 × CH_2_ClCH_2_Cl is also considered (i.e., EXP3, equivalent ATTREX quantity shown with open stars).

Figure 1 shows the simulated time‐varying abundance of CH_2_Cl_2_, CHCl_3_, and C_2_Cl_4_ at the level of zero radiative heating (LZRH, ∼15 km, ∼360K potential temperature) in the TTL – above which air ascends into the stratosphere [e.g., *Gettelman et al.*, 2004]. Long‐term aircraft [*Leedham Elvidge et al.*, 2015] and surface observations [*Hossaini et al.*, 2015] of CH_2_Cl_2_ have shown a rapid increase in its tropospheric abundance in the last decade, particularly in the Northern Hemisphere (NH), owing to industrial sources [*Carpenter et al.*, 2014; *Hossaini et al.*, 2015]. This information is contained in the model forcing data and, as shown in Figure 1, caused a largely monotonic increase in the stratospheric injection of CH_2_Cl_2_ over the simulation period. At the LZRH, CH_2_Cl_2_ increased from, on average, 18 ppt in 2005 to 33 ppt in 2013, i.e., an increase of 83%.

Figure 1 also shows high‐altitude CH_2_Cl_2_ observations from the NASA Airborne Tropical Tropopause Experiment (ATTREX). Whole air samples were collected on board the unmanned Global Hawk aircraft during deployment in 2011 and 2013 and were analyzed by the University of Miami using gas chromatography/mass spectrometry. These observations provide a valuable consistency check of the model. The agreement between modeled and observed CH_2_Cl_2_ is excellent. For CHCl_3_, the model exhibits a low bias of ∼15% compared to the 2013 observations. Due to no significant long‐term surface CHCl_3_ trend, the stratospheric SGI of CHCl_3_ varied relatively little over the simulation. C_2_Cl_4_ is a relatively minor contributor to chlorine SGI and its tropospheric abundance has steadily declined in the last decade [*Montzka et al.*, 2011]. Both model and observation indicate <1 ppt of this VSLS at the LZRH in 2013.

We estimate that the stratospheric SGI of CHCl_3_, CH_2_Cl_2_, and C_2_Cl_4_ provided a total of 87 ppt Cl in 2013. This agrees well (to within 5%) of the same quantity derived from ATTREX observations (∼91 ppt Cl). Consideration of C_2_HCl_3_ and CH_2_ClCH_2_Cl (i.e., EXP3) increases the model estimate to ∼102 ppt Cl. The observed chlorine SGI is also greater when these gases are included; shown in Figure 1d, as the difference between the filled and open stars. This difference is virtually entirely due to CH_2_ClCH_2_Cl. Due to a particularly short local tropospheric lifetime (typically several days) and a low tropospheric abundance (< 1 ppt), C_2_HCl_3_ makes a negligible contribution to chlorine SGI. We find that CH_2_Cl_2_, CHCl_3_, C_2_Cl_4_, and CH_2_ClCH_2_Cl account for 65%, 17%, 3.5%, and 14.5% of the total SGI of chlorine in 2013, respectively. Local lifetimes of these VSLS in the tropical boundary layer are estimated in the range 98–133, 100–136, 60–81, and 42–58 days, respectively [*Carpenter et al.*, 2014]. The contribution of CH_2_ClCH_2_Cl could be larger as the model underestimates measured values in 2013. Long‐term observations of this VSLS are needed to constrain its tropospheric abundance.

Our modeled estimate of stratospheric chlorine from the SGI of VSLS in 2013 is 30% larger than that reported by *Carpenter et al.* [2014], due to the continued increases in CH_2_Cl_2_ mixing ratios. Note that the ocean‐emitted VSLS CHBr_2_Cl, CH_2_BrCl, and CHBrCl_2_ are not included in our estimate, as the focus of this work is on anthropogenic VSLS. Besides, their total SGI is estimated to provide <0.4 ppt Cl [*Carpenter et al.*, 2014] and is therefore very small compared to the species considered here.

### 3.2 Product Gas Injection

**Figure 2:**
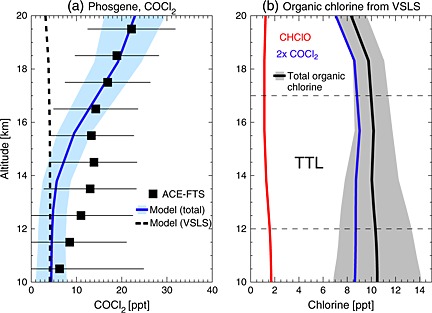
(a) Observed tropical mean profile of COCl_2_ volume mixing ratio (ppt) from the ACE‐FTS satellite instrument (2006–2012 average). The horizontal bars denote ±1 standard deviation. Also shown are corresponding model profiles of total COCl_2_ (from EXP3, solid line, shading ±1 standard deviation) and VSLS‐derived COCl_2_ (from EXP3 minus the control run, dashed line). (b) Simulated tropical mean profiles of organic chlorine from VSLS‐derived COCl_2_, CHClO, and their total (shading ±1 standard deviation) in 2013. The location of the tropical tropopause layer (TTL) is indicated.

Organic products derived from VSLS degradation include halogenated peroxy radicals, hydroperoxides, peroxynitrates, and carbonyl compounds [e.g., *Ko et al.*, 2003; *Hossaini et al.*, 2010]. We found that the only organic products simulated with tropospheric mixing ratios >1 ppt were (i) phosgene, COCl_2_, formed principally from CHCl_3_ (with minor contributions from C_2_Cl_4_ and C_2_HCl_3_) and (ii) formyl chloride, CHClO, formed from CH_2_Cl_2_. Upper tropospheric COCl_2_ measurements (Figure 2) are made by the Atmospheric Chemistry Experiment Fourier transform spectrometer (ACE‐FTS) on board the SCISAT satellite [e.g., *Fu et al.*, 2007; *Brown et al.*, 2011]. Here we use the v3.0 data product for measurements between 2006 and September 2010, and v3.5 for October 2010 to 2012.

Throughout the observed profile shown in Figure 2a, model‐derived COCl_2_ is within the measurement variability at all altitudes. In the tropical upper troposphere (∼10 km), VSLS account for virtually all simulated COCl_2_ and the agreement between model and observation is good. In the lower part of the TTL, however, model COCl_2_ exhibits a low bias. Potential explanations for this include a missing source (e.g., from a VSLS not considered) or an underestimate of the phosgene yield from C_2_Cl_4_ or C_2_HCl_3_. The former seems unlikely as the most abundant chlorinated VSLS known to produce phosgene, i.e., CHCl_3_, is included, though the underestimate of CHCl_3_, shown in Figure 1 (albeit small) may contribute to the low COCl_2_ bias. Alternatively, a dynamical influence may include an underestimate of the in‐mixing of phosgene‐containing air from the extratropical lower stratosphere into the TTL.

Systematic errors in the ACE‐FTS COCl_2_ mixing ratios are dominated by spectroscopic errors, assumed ∼30% [*Fu et al.*, 2007]; this results from the lack of hot bands in the COCl_2_ spectroscopic line list and uncertainties in line intensities. It is therefore particularly challenging to assess the fidelity of simulated COCl_2_ in the TTL. We note that around the LZRH (∼15 km), model COCl_2_ agrees well with the observed values and here VSLS account for ∼95% of total COCl_2_. Above 20 km, COCl_2_ production is dominated by the long‐lived solvents CCl_4_ and CH_3_CCl_3_.

To our knowledge, no atmospheric observations of the second most abundant product gas, CHClO, exist. Its simulated surface mixing ratio in the NH, where its primary source in the model (CH_2_Cl_2_) is most abundant, is ∼3–5 ppt (see supporting information). Figure 2b shows that CHClO provides a small, though previously unidentified, contribution to PGI of ∼2 ppt Cl.

**Figure 3:**
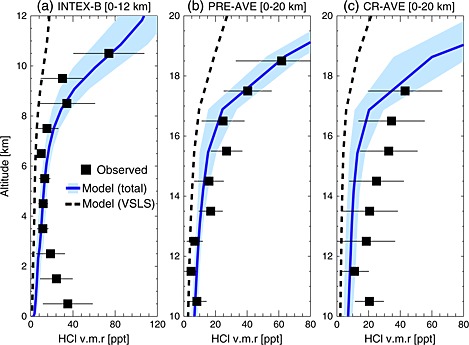
Observed profiles of HCl volume mixing ratio (ppt) from the NASA (a) 2006 INTEX‐B mission, (b) 2004 Pre‐AVE mission, and (c) 2006 CR‐AVE mission. INTEX‐B measurements obtained on board the DC‐8 aircraft (0–12 km) between ∼40°N and 61°N latitude (Anchorage deployment, Alaska). Pre‐AVE and CR‐AVE measurements obtained on board the WB‐57 aircraft (10–20 km) within the latitude ranges ∼3°S to ∼8°N and ∼1°S to ∼20°N. Measured HCl profiles are campaign means calculated in 1 km altitude bins. Horizontal lines on observed data denote ±1 standard deviation. Also shown are corresponding model mean profiles of HCl derived from all sources (solid profile, ±1 standard deviation) and HCl derived from anthropogenic VSLS only (dashed line).

In addition to organic products, VSLS‐derived HCl may also enter the stratosphere through PGI. In situ measurements of tropospheric HCl are generally sparse, though measured profiles are available from previous NASA aircraft missions [*Marcy et al.*, 2007, 2004; *Kim et al.*, 2008], shown in Figure 3, and more recent aircraft sampling around the tropopause [*Jurkat et al.*, 2014]. In the marine boundary layer, sea salt is the dominant chlorine source [e.g., *Saiz‐Lopez and von Glasow*, 2012]. Here simulated HCl exhibits a low bias against observed values as sea salt chlorine is not considered (as not relevant for the stratosphere). However, in the free troposphere and TTL—where chlorine is mostly derived from the breakdown of organic source gases—agreement between simulated and observed HCl is generally good. In 2013, VSLS accounted for, on average, ∼40% of total HCl around the LZRH (∼9 ppt of VSLS‐derived HCl in absolute terms), with the remainder supplied from a combination of stratospheric‐influenced air and the in situ oxidation of the relatively long‐lived source gas CH_3_Cl [e.g., *Marcy et al.*, 2007].

By summing the modeled organic and inorganic contributions, we estimate a total stratospheric PGI of ∼21 ppt Cl in 2013 (EXP3). This value falls within the estimated range of 0–50 ppt Cl reported by *Carpenter et al.* [2014] but is slightly lower than their best estimate of 25 ppt Cl. This difference is largely due to the lower PGI contribution from COCl_2_ from the model relative to the estimate of *Carpenter et al.* [2014], as summarized in Table 1. Note that our total PGI estimate also includes a contribution from CHClO which has been previously unquantified. Our analysis shows, for the first time, that PGI due to VSLS‐derived products constitutes a nonzero supply of chlorine to the stratosphere. However, further insight into the impact of mechanistic and kinetic uncertainties in the chemical mechanism employed is required, beyond the scope of this work, in order to fully constrain the derived chlorine budget. In particular, this includes a need for knowledge of the aqueous phase processing of soluble product gases (e.g., COCl_2_, CHClO) and heterogeneous processes occurring on, for example, cirrus ice particles within the TTL [e.g., *von Hobe et al.*, 2011].

**Table 1:** Simulated Source and Product Gas Contributions to Total Stratospheric Chlorine Supplied From VSLS (ppt Cl)^a^

	2005	2006	2007	2008	2009	2010	2011	2012	2013	WMO 2014^c^
Source Gas Sum^b^	57.3	59.0	62.4	64.6	63.8	70.3	70.9	75.2	87.4 [102.3]	72(50–95)
Phosgene	8.0	7.7	8.1	8.8	7.9	9.0	8.6	8.9	8.3 [9.1]	15(0–30)
Formyl Chloride	1.1	1.1	1.3	1.3	1.4	1.6	1.5	1.6	2.4 [2.4]	Not considered
Hydrogen Chloride	3.0	2.9	3.2	3.6	3.0	3.3	3.3	3.1	7.6 [9.4]	10(0–20)
Product Gas Sum	12.1	11.8	12.7	13.8	12.3	13.9	13.5	13.7	18.4 [20.9]	25(0–50)
TOTAL Cl	69.4	70.8	75.1	78.4	76.1	82.4	84.4	88.9	105.8 [123.2]	95(50–145)

a Model estimates are reported from EXP2. For 2013, values are also reported for EXP3 (square brackets). Estimates from the 2014 WMO/UNEP Scientific Assessment of Ozone Depletion are compared to the simulated values from this work.

b Sum of (3 × CHCl_3_) + (2 × CH_2_Cl_2_) + (4 × C_2_Cl_4_); with additional contribution of (3 × C_2_HCl_3_) and (2 × CH_2_ClCH_2_Cl) (see data in square brackets).

c Best estimate and range reported in WMO/UNEP Scientific Assessment of Ozone Depletion 2014 [*Carpenter et al.*, 2014].

### 3.3 Total Chlorine

We estimate the total chlorine injection from anthropogenic VSLS ( ${\text{Cl}}_{y}^{\text{VSLS}}$)—the sum of SGI and PGI contributions—was 123 ppt Cl in 2013 (EXP3, Table 1). SGI accounts for ∼83% of total ${\text{Cl}}_{y}^{\text{VSLS}}$ with the remainder supplied from PGI of VSLS‐derived COCl_2_ (7%), CHClO (2%), and HCl (8%). Our simulated ${\text{Cl}}_{y}^{\text{VSLS}}$ is larger than the best estimate of 95 ppt Cl reported by *Carpenter et al.* [2014], owing to a larger SGI contribution and the continued increase in CH_2_Cl_2_ mixing ratios. However, it falls within their reported 50–145 ppt Cl range.

To estimate the purely anthropogenic component of stratospheric ${\text{Cl}}_{y}^{\text{VSLS}}$, a further sensitivity experiment (EXP4) was performed, identical to EXP3, but with surface CH_2_Cl_2_ and CHCl_3_ reduced by 10% and 75%, respectively (to remove the estimated natural component of emissions). C_2_Cl_4_, C_2_HCl_3_, and CH_2_ClCH_2_Cl are thought to be exclusively anthropogenic and were therefore unchanged. From EXP4 we estimate ∼80% of the simulated stratospheric ${\text{Cl}}_{y}^{\text{VSLS}}$ in 2013 is attributable to anthropogenic activity.

**Figure 4:**
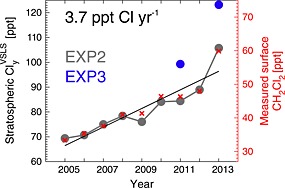
Time series of simulated annual mean total stratospheric chlorine from VSLS (sum of source and product gas contributions, filled circles, left axis). A linear trend line is applied to EXP2 which considered CH_2_Cl_2_, CHCl_3_, and C_2_Cl_4_, and the 2005–2013 mean growth rate (ppt Cl^−1^) is annotated. EXP3 (blue) also considered C_2_HCl_3_ and CH_2_ClCH_2_Cl. Also overlaid are annual mean CH_2_Cl_2_ mixing ratios (ppt) in the Northern Hemisphere calculated from NOAA surface observations (red crosses, right axis).

Based on EXP2, as it contained the same VSLS throughout the simulation, ${\text{Cl}}_{y}^{\text{VSLS}}$ increased by ∼52% over the 2005–2013 period (Table 1). A linear fit to the data indicates a mean growth rate of ∼3.7 ppt Cl yr^−1^ (Figure 4), driven by the ongoing increase in the surface concentration of CH_2_Cl_2_. In addition to causing an enhanced SGI, CH_2_Cl_2_ growth has also enhanced PGI; both CHClO (derived from CH_2_Cl_2_) and HCl exhibited a sharp increase over the 2012–2013 period, coinciding with a particularly sharp increase in surface CH_2_Cl_2_ and indeed total ${\text{Cl}}_{y}^{\text{VSLS}}$ (Figure 4). Between 2012 and 2013, ${\text{Cl}}_{y}^{\text{VSLS}}$ increased by ∼20%.

## 4 Concluding Remarks

Knowledge of the stratospheric loading of ozone‐depleting substances and their trends is required to predict the future evolution of stratospheric O_3_ and recovery of the ozone layer. Constraining the supply of chlorine from chemicals not regulated by the Montreal Protocol is particularly important. Here, using a global model supported by atmospheric observations, we show that the contribution of anthropogenic VSLS has increased significantly in the last decade. A 2013 stratospheric ${\text{Cl}}_{y}^{\text{VSLS}}$ loading of >100 ppt is large enough to significantly impact O_3_ loss rates in the lower stratosphere [*Hossaini et al.*, 2015], where surface temperature and climate are particularly sensitive to O_3_ perturbations. Indeed, trends in anthropogenic chlorine VSLS are suggested to already have contributed a nonzero amount to the radiative forcing of climate since the preindustrial era [*Hossaini et al.*, 2015].

Although ${\text{Cl}}_{y}^{\text{VSLS}}$ remains small in comparison to the total stratospheric chlorine load from long‐lived ozone‐depleting substances (>3000 ppt Cl), it is far larger than that supplied from newly detected chlorofluorocarbons and hydrochlorofluorocarbons (<10 ppt Cl) [*Laube et al.*, 2014]. Further, ${\text{Cl}}_{y}^{\text{VSLS}}$ will continue to increase if the observed growth in tropospheric CH_2_Cl_2_ continues. This could likely be the case if the upward CH_2_Cl_2_ trend is attributable to the continued industrialization of developing countries and/or release following its use as a feedstock in the production of HFCs, the second generation of CFC replacement gases, for which a global market expansion seems likely in the coming years, or indeed other chemicals.

## Supporting information



Texts S1 and S2, Tables S1–S9, and Figures S1–S3
