# Supplementary material for: Growth in stratospheric chlorine from short‐lived chemicals not controlled by the Montreal Protocol
Source: Geophys Res Lett. 2015 Jun 1;42(11):4573–80. doi: 10.1002/2015GL063783 (PMC4981078; doi:10.1002/2015GL063783)
Supplement: Supplementary file 1 — Texts S1 and S2, Tables S1–S9, and Figures S1–S3 [file GRL-42-4573-s001.docx]

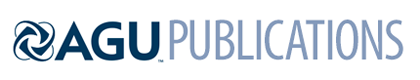


*Geophysical Research Letters*

Supporting Information for

**Growth in stratospheric chlorine from short-lived chemicals not controlled by the Montreal Protocol**

R. Hossaini^1^, M. P. Chipperfield^1^, A. Saiz-Lopez^2^, J. J. Harrison^3^, R. von Glasow^4^, R. Sommariva^4,*^, E. Atlas^5^, M. Navarro^5^, S. A. Montzka^6^, W. Feng^1^, S. Dhomse^1^, C. Harth^7^, J. Mühle^7^, C. Lunder^8^, S. O’Doherty^9^, D. Young^9^, S. Reimann^10^, M. K. Vollmer^10^, P. B. Krummel^11^, and P. F. Bernath^12^

^1^ School of Earth and Environment, University of Leeds, Leeds, UK.

^2^ Atmospheric Chemistry and Climate Group, Institute of Physical Chemistry Rocasolano, CSIC, Spain.

^3^ National Centre for Earth Observation, Department of Physics and Astronomy, University of Leicester, Leicester, UK.

^4^ Centre for Ocean and Atmospheric Sciences, School of Environmental Sciences, University of East Anglia, UK.

^5^ Rosenstiel School of Marine and Atmospheric Science, University of Miami, USA.

^6^ National Ocean and Atmospheric Administration, Boulder, USA.

^7^ Scripps Institution of Oceanography, University of California, San Diego, USA.

^8^ Monitoring and Information Technology Department, Norwegian Institute for Air Research, Kjeller, Norway.

^9^ Atmospheric Chemistry Research Group, School of Chemistry, University of Bristol, Bristol, UK.

^10^ Empa, Swiss Federal Laboratories for Materials Science and Technology, Dübendorf, Switzerland.

^11^ CSIRO Oceans & Atmosphere Flagship, Aspendale, Victoria, Australia.

^12^ Department of Chemistry and Biochemistry, Old Dominion University, Norfolk, Virginia, USA.

*now at: Department of Chemistry, University of Leicester, Leicester, UK.

**Contents of this file**

| Text S1  Table S1  Table S2  Figure S1  Figure S2  Table S3  Table S4  Table S5  Table S6  Text S2  Tables S7-S9  Figure S3 | Description of chlorine chemistry scheme  Summary of source gases  Summary of product gases  CHCl_3_ degradation scheme  CH_2_Cl_2_ degradation scheme  List of reactions (CHCl_3_ degradation)  List of reactions (CH_2_Cl_2_ degradation)  List of reactions (other chlorine source gases)  List of reactions (inorganic chlorine)  Description of surface measurements  Measured surface mixing ratios of chlorine source gases  Latitude-pressure cross section of CHClO |
| --- | --- |

**Introduction**

This Supporting Information contains a description of the model chlorine chemistry (Text S1). A summary of model chlorine source gases (Table S1) and product gases (Table S2) is given. A list of reactions is given in Tables S3-S6. Figures S1 and S2 depict the degradation schemes of CHCl_3_ & CH_2_Cl_2_, respectively. The measured surface mixing ratios of chlorine very short-lived substances, used as a model boundary condition (Text S2), are summarized in Tables S7-S9. Figure S3 shows the modelled tropospheric distribution of CHClO.

**Text S1 : Chlorine Chemistry Scheme in the TOMCAT Model**

The TOMCAT model configuration here contains 11 chlorine source gases, including long-lived substances (lifetimes >6 months), anthropogenic VSLS (Table S1, lifetimes <6 months with a significant/predominate anthropogenic source) & natural VSLS (lifetimes <6 months, predominately oceanic).

**Degradation of CHCl_3_**

The degradation mechanism of CHCl_3_ is based on the general halocarbon scheme outlined in Ko and Poulet et al. [2003]. The scheme considers 10 organic products (Figure S1). Tropospheric loss of CHCl_3_ is dominated by OH-initiated oxidation. This initial step proceeds via hydrogen abstraction resulting in a CCl_3_ radical. Under tropospheric conditions, CCl_3_ is rapidly oxidized (seconds) [e.g. Jowko et al., 2003; Brudnik et al., 2008] forming a peroxy radical (CCl_3_O_2_) which in turn may react with NO, NO_2_, HO_2_, CH_3_O_2_ or itself [e.g. Simonaitis and [Heicklen](http://www.sciencedirect.com/science/article/pii/0009261479807447), 1979; Catoire et al., 1996]. The expected organic products of CHCl_3_ degradation include chlorinated peroxynitrates, hydroperoxides, alcohols and carbonyl compounds. The relative yield of these products depends on background loadings NO_x_ and HO_x_. The expected major organic product is phosgene (COCl_2_). Table S3 summarizes the reactions & kinetic data.

**Degradation of CH_2_Cl_2_**

The CH_2_Cl_2_ degradation scheme is also based on the general halocarbon mechanism outlined in Ko and Poulet et al. [2003] and shares a number of similarities with the CHCl_3_ scheme. The scheme considers 10 organic products (Figure S2). The reactions of CHCl_2_O_2_, CHCl_2_OOH, CHCl_2_O_2_NO_2_, CHCl_2_OH, COCl_2_ and CHClO (also in the CHCl_3_ scheme) are given in Table S3. Reactions of the unique species CH_2_ClO_2_, CH_2_ClOOH, CH_2_ClO_2_NO_2_ and CH_2_ClOH are given in Table S4. Tropospheric loss of CH_2_Cl_2_ is dominated by OH-initiated oxidation. The expected major organic product of CH_2_Cl_2_ degradation is formyl chloride (CHClO).

**Other Chlorinated Source Gases**

In addition to CHCl_3_ and CH_2_Cl_2_, the relatively minor anthropogenic chlorinated VSLS C_2_Cl_4_, C_2_HCl_3_ and CH_2_ClCH_2_Cl are also considered. To avoid the large computational expense of adding a full degradation scheme for these gases and, also due to mechanistic uncertainties, a simplified treatment of their chemical loss is employed. We assume upon degradation (i.e. due to OH-initiated oxidation / photolysis, explicitly calculated) a phosgene yield (see Table S5) based on laboratory estimates [Tuazon et al., 1988; Kindler et al., 1995]. Note, this approach was also used for the two long-lived (stratospheric) phosgene sources in the model (CCl_4_ and CH_3_CCl_3_). Other chlorinated source gases (i.e. CH_3_Cl, CHBr_2_Cl, CH_2_BrCl and CHBrCl_2_) released Cl atoms instantaneously upon degradation. Reactions of inorganic product gases are summarized in Table S6.

**Table S1:** Summary of chlorine-containing source gases in the model

| **Tracer #** | **Source Gas** | **Formula** | **Type** |
| --- | --- | --- | --- |
| 1 | Methyl chloride | CH_3_Cl | Long-lived |
| 2 | Carbon tetrachloride | CCl_4_ | Long-lived |
| 3 | Methyl chloroform | CH_3_CCl_3_ | Long-lived |
| 4 | Chloroform | CHCl_3_ | Anthropogenic VSLS |
| 5 | Dichloromethane | CH_2_Cl_2_ | Anthropogenic VSLS |
| 6 | Tetrachloroethene | C_2_Cl_4_ | Anthropogenic VSLS |
| 7 | Trichloroethene | C_2_HCl_3_ | Anthropogenic VSLS |
| 8 | 1,2-dichloroethane | CH_2_ClCH_2_Cl | Anthropogenic VSLS |
| 9 | Dibromochloromethane | CHBr_2_Cl | Natural VSLS |
| 10 | Bromodichloromethane | CHBrCl_2_ | Natural VSLS |
| 11 | Bromochloromethane | CH_2_BrCl | Natural VSLS |

**Table S2:** Summary of chlorine-containing product gases in the model

| Organic | CCl_3_O_2_, CHCl_2_O_2_, CH_2_ClO_2_, CCl_3_OOH, CHCl_2_OOH, CH_2_ClOOH, CCl_3_O_2_NO_2_, CHCl_2_O_2_NO_2_, CH_2_ClO_2_NO_2_, CCl_3_OH, CHCl_2_OH, CH_2_ClOH, COCl_2_, CHClO |
| --- | --- |
| Inorganic (Cl_y_) | Cl, ClO, Cl_2_, HCl, HOCl, ClNO_2_, ClONO_2_, OClO, BrCl |

**Figure S1.** CHCl_3_ Degradation Scheme. Red boxes show 11 chlorocarbons explicitly treated (1 source gas & 10 product gases). Reactions assumed instantaneous are shown with blue arrows (for kinetic justification see, for example, Jowko et al. 2003; Brudnik et al. 2008; Biggs et al. 1999; Hou et al. 2005; Catoire et al. 1996). Cl atom release shown in green. Species wet deposited marked with asterisk. Henry’s Law constants and solubility of chlorinated organic products were assumed equal to analogous brominated compounds [Krysztofiak et al., 2012].

**Figure S2.** As Figure S1 but for CH_2_Cl_2_.

**Table S3:** Reactions involved in the degradation of CHCl_3_

| **#** | **Reaction** | **Rate Constant**  **(k, cm^3^ molec^-1^ s^-1^ unless noted)** | **Comments** | **Reference** |
| --- | --- | --- | --- | --- |
| **1** | CHCl_3_ + OH (+O_2_) --- CCl_3_O_2_ +H_2_O | k(T) = 2.2E-12.exp(-920/T) | - | JPL |
| **2** | CHCl_3_ + Cl (+O_2_) ---- CCl_3_O_2_ + HCl | k(T) = 3.3E-12.exp(-990/T) | - | JPL |
| **3** | CHCl_3_ + hv (+O_2_) --- CHCl_2_O_2_ + Cl | - | Calculated from abs. cross section | JPL |
| **4** | CCl_3_O_2_ + NO_2_ (+M) --- CCl_3_O_2_NO_2_ | k_0_(T) = 9.2E-29(T/298)^-6.0^ [N_2_]  k_∞_(T) = 1.5E-12(T/298)^-0.7^ | Termolecular | IUPAC |
| **5** | CCl_3_O_2_ + NO --- COCl_2_ + NO_2_ + Cl | k(T) = 7.3E-12.exp(270/T) | - |  |
| **6a** | CCl_3_O_2_ + HO_2_ --- CCl_3_OOH + O_2_ | k(T) = 4.7E-13.exp(710/T) | Branch 1 | IUPAC |
| **6b** | CCl_3_O_2_ + HO_2_ --- COCl_2_ + OH + O_2_ + Cl | k(T) = 4.7E-13.exp(710/T) | Branch 0 | IUPAC |
| **7a** | CCl_3_O_2_ + CH_3_O_2_ --- COCl_2_ + Cl + CH_3_O + O_2_ | k(298 K) = 6.6E-12 | Branch 0.5 | IUPAC |
| **7b** | CCl_3_O_2_ + CH_3_O_2_ --- CCl_3_OH + CH_2_O + O_2_ | k(298 K) = 6.6E-12 | Branch 0.5 | IUPAC |
| **8** | CCl_3_O_2_ + CCl_3_O_2_ --- 2COCl_2_ + 2Cl + O_2_ | k(T) = 3.3E-13.exp(740/T) | Self-reaction | IUPAC |
| **9** | CHCl_2_O_2_ + NO_2_ (+M) --- CHCl_2_O_2_NO_2_ | - | Assumed analogous to R4 | - |
| **10** | CHCl_2_O_2_ + NO --- CHClO + NO_2_ + Cl | k(T) = 4.0E-12.exp(360/T) | - | MCM |
| **11a** | CHCl_2_O_2_ + HO_2_ --- CHCl_2_OOH + O_2_ | k(T) = 5.6E-13.exp(700/T) | Branch 0.7 | IUPAC |
| **11b** | CHCl_2_O_2_ + HO_2_ --- CHClO + OH + O_2_ + Cl | k(T) = 5.6E-13.exp(700/T) | Branch 0 | IUPAC |
| **11c** | CHCl_2_O_2_ + HO_2_ --- COCl_2_ + H_2_O + O_2_ | k(T) = 5.6E-13.exp(700/T) | Branch 0.3 | IUPAC |
| **12a** | CHCl_2_O_2_ + CH_3_O_2_ --- CHClO + Cl + CH_2_O + HO_2_ | k(298 K) = 8.8E-12 | Branch 0.7 | Biggs et al. [1999] |
| **12b** | CHCl_2_O_2_ + CH_3_O_2_ --- CHCl_2_OH + CH_2_O + O_2_ | k(298 K) = 8.8E-12 | Branch 0.3 | Biggs et al. [1999] |
| **13** | CHCl_2_O_2_ + CHCl_2_O_2_ --- 2CHClO + 2Cl + O_2_ | k(298 K) = 7.0E-12 | Self-reaction | Biggs et al. [1999] |
| **14** | CCl_3_O_2_NO_2_ (+M) --- CCl_3_O_2_ + NO_2_ | k_0_(T) = 4.3E-3.exp(-10235/T)[N_2_] s^-1^  k∞(T) = 4.8E16(-11820/T) s^-1^ | Decomposition | IUPAC |
| **15** | CCl_3_O_2_NO_2_ + hv --- COCl_2_ + Cl + NO_3_ | - | Assumed CH_3_O_2_NO_2_ cross section | (JPL) |
| **16** | CHCl_2_O_2_NO_2_ (+M) --- CHCl_2_O_2_ + NO_2_ | - | Assumed analogous to R14 | - |
| **17** | CHCl_2_O_2_NO_2_ + hv --- CHClO + Cl + NO_3_ | - | Assumed CH_3_O_2_NO_2_ cross section | (JPL) |
| **18** | CCl_3_OOH + OH --- CCl_3_O_2_ + H_2_O | k(T) =1.9E-12.exp(190/T) | - | MCM |
| **19** | CCl_3_OOH + hv --- COCl_2_ + Cl + OH | - | Assumed CH_3_OOH cross section | (JPL) |
| **20** | CHCl_2_OOH + OH --- CHCl_2_O_2_ + H_2_O | k(T) =1.9E-12.exp(190/T) | - | MCM |
| **21** | CHCl_2_OOH + hv --- CHClO + Cl + OH | - | Assumed CH_3_OOH cross section | (JPL) |
| **22** | CCl_3_OH + OH --- COCl_2_ + Cl + H_2_O | k = 3.6E-14 | - | MCM |
| **23** | CHCl_2_OH + OH (+O_2_) --- COCl_2_ + HO_2_ + H_2_O | k = 9.34E-13 | - | MCM |
| **24** | COCl_2_ + OH --- CO + OH + 2Cl | k = 5.0E-15 | - | IUPAC |
| **25** | COCl_2_ + O(^1^D) --- CO_2_ + 2Cl | k(T) = 2.2E-11.exp(30/T) | - | JPL |
| **26** | COCl_2_ + hv --- CO + 2Cl | - | Calculated from abs. cross section | JPL |
| **27** | CHClO + OH --- Cl + CO + H_2_O | k = 5.0E-13 | - | IUPAC |
| **28** | CHClO + Cl --- Cl + HCl + CO | k(T) = 1.2E-11.exp(-820/T) | - | Ko & Poulet et al. [2003] |
| **29** | CHClO + hv --- Cl + CO + HO_2_ | - | Calculated from abs. cross section | JPL |

**Notes:** Jet Propulsion Laboratory (JPL, Sander et al. 2011). International Union of Pure & Applied Chemistry (IUPAC, Atkinson et al., 2008 or http://iupac.pole-ether.fr/). Master Chemical Mechanism (MCM, http://mcm.leeds.ac.uk/MCM/).

**Table S4:** Reactions involved in the degradation of CH_2_Cl_2_

| **#** | **Reaction** | **Rate Constant**  **(k, cm^3^ molec^-1^ s^-1^ unless noted)** | **Comments** | **Reference** |
| --- | --- | --- | --- | --- |
| **30** | CH_2_Cl_2_ + OH (+O_2_) --- CHCl_2_O_2_ + H_2_O | k(T) = 1.9E-12.exp(-870/T) | - | JPL |
| **31** | CH_2_Cl_2_ + Cl (+O_2_) --- CHCl_2_O_2_ + HCl | k(T) = 7.4E-12.exp(-910/T) | - | JPL |
| **32** | CH_2_Cl_2_ + hv (+O_2_) --- CH_2_ClO_2_ + Cl | - | Calculated from abs. cross section | JPL |
| **33** | CH_2_ClO_2_ + NO_2_ (+M) --- CH_2_ClO_2_NO_2_ | - | Assumed analogous to R4 | - |
| **34** | CH_2_ClO_2_ + NO --- (+O_2_) --- CHClO + HO_2_ + NO_2_ | k(T) = 7.0E-12.exp(300/T) | - |  |
| **35a** | CH_2_ClO_2_ + HO_2_ --- CH_2_ClOOH + O_2_ | k(T) = 3.2E-13.exp(820/T) | Branch 0.3 | IUPAC |
| **35b** | CH_2_ClO_2_ + HO_2_ --- CHClO + O_2_ + H_2_O | k(T) = 3.2E-13.exp(820/T) | Branch 0.7 | IUPAC |
| **36a** | CH_2_ClO_2_ + CH_3_O_2_ (+O_2_) --- CHClO + CH_3_O + HO_2_ + O_2_ | k = 2.5E-12 | Branch 0.66 | IUPAC |
| **36b** | CH_2_ClO_2_ + CH_3_O_2_ --- CH_2_ClOH + CH_2_O + O_2_ | k = 2.5E-12 | Branch 0.34 | IUPAC |
| **37** | CH_2_ClO_2_ + CH_2_ClO_2_ (+2O_2_) --- 2CHClO + O_2_ + 2HO_2_ | k(T) = 1.9E-13.exp(870/T) | - | IUPAC |
| **38** | CH_2_ClO_2_NO_2_ (+M) --- CH_2_ClO_2_ + NO_2_ | - | Assumed analogous to R14 | - |
| **39** | CH_2_ClO_2_NO_2_ + hv --- Cl + CH_2_O + NO_3_ | - | Assumed CH_3_O_2_NO_2_ cross section | (JPL) |
| **40** | CH_2_ClOOH + OH --- CH_2_ClO_2_ + H_2_O | k(T) = 1.9E-12.exp(190/T) | - | MCM |
| **41** | CH_2_ClOOH + hv --- CHClO + OH | - | Assumed CH_3_OOH cross section | (JPL) |
| **42** | CH_2_ClOH + OH --- CHClO + HO_2_ | k = 1.8E-12 | - | MCM |

**Table S5:** Reactions involved in the degradation of other chlorinated source gases

| **#** | **Reaction** | **Rate Constant**  **(k, cm^3^ molec^-1^ s^-1^ unless noted)** | **Comments** | **Reference** |
| --- | --- | --- | --- | --- |
| **43** | C_2_Cl_4_ + OH --- COCl_2_ + products | k(T) = 3.5E-12.exp(-920/T) | COCl_2_ yield of 0.5 | IUPAC; Kindler et al. [1995]; Tuazon et al. [1988] |
| **44** | C_2_Cl_4_ + Cl | k_0_ (296 K) = 1.8E-28, F_c_ = 0.6  k_∞_ (296 K)= 4.0E-11 | COCl_2_ yield of 0.35 | Thüner et al. [1999] |
| **45** | C_2_HCl_3_ + OH --- COCl_2_ + products | k(T) = 3.0E-13.exp(565/T) | COCl_2_ yield of 0.4 | IUPAC; Kindler et al. [1995]; Tuazon et al. [1988] |
| **46** | CH_2_ClCH_2_Cl + OH --- 2Cl + products | k(T) = 8.69E-12(-1070/T) | - | MCM |
| **47** | CH_2_ClCH_2_Cl + hv --- 2Cl + products | - | Assumed CH_2_Cl_2_ cross section | JPL |
| **48** | CHBr_2_Cl + OH --- 2Br + Cl | k(T) = 9.0E-13(-423/T) | - | Orkin et al. [2013] |
| **49** | CHBr_2_Cl + hv --- 2Br + Cl | - | Calculated from abs. cross section | JPL |
| **50** | CHBrCl_2_ + OH --- 2Cl + Br | k(T) = 9.4E-13(-513/T) | - | Orkin et al. [2013] |
| **51** | CHBrCl_2_ + hv --- 2Cl + Br | - | Calculated from abs. cross section | JPL |
| **52** | CH_2_BrCl + OH --- Br + Cl | k(T) = 2.4E-12.exp(-920) | - | JPL |
| **53** | CH_2_BrCl + hv --- Br + Cl | - | Calculated from abs. cross section | JPL |
| **54** | CH_3_Cl + OH --- Cl + H_2_O | k(T) = 2.4E-12.exp(-1250/T) | - | JPL |
| **55** | CH_3_Cl + Cl --- Cl + HCl | k(T) = 2.17E-11.exp(-1130/T) | - | JPL |
| **56** | CH_3_Cl + hv --- Cl | - | Calculated from abs. cross section | JPL |
| **57** | CCl_4_ + O(^1^D) --- COCl_2_ + products | k = 3.3E-10 | COCl_2_ yield of 1 | JPL; Kindler et al. [1995]] |
| **58** | CCl_4_ + hv --- COCl_2_ + products | Calculated from abs. cross section | COCl_2_ yield of 1 | JPL |
| **59** | CH_3_CCl_3_ + OH --- COCl_2_ + products | k(T) = 1.64E-12.exp(-1520/T) | COCl_2_ yield of 1 | JPL |
| **60** | CH_3_CCl_3_ + Cl --- COCl_2_ + products | k(T) = 3.23E-12.exp(-1770/T) | COCl_2_ yield of 1 | JPL |
| **61** | CH_3_CCl_3_ + hv --- 3Cl | Calculated from abs. cross section | Calculated from abs. cross section | JPL |

**Table S6:** Gas-phase inorganic chlorine reactions

| **#** | **Reaction** | **Rate Constant**  **(k, cm^3^ molec^-1^ s^-1^ unless noted)** | **Comments** | **Reference** |
| --- | --- | --- | --- | --- |
|  | **Bimolecular** |  |  |  |
| **61** | Cl + O_3_ --- ClO + O_2_ | k(T) = 2.3E-11.exp(-200/T) | - | JPL |
| **62** | ClO + HO_2_ --- HOCl + O_2_ | k(T) = 2.6E-12.exp(290/T) | - | JPL |
| **63** | Cl + HO_2_ --- HCl + O_2_ | k(T) = 1.4E-11.exp(270/T) | - | JPL |
| **64** | HCl + OH --- Cl + H_2_O | k(T) = 1.8E-12.exp(-250/T) | - | JPL |
| **65** | ClO + NO --- Cl + NO2 | k(T) = 6.4E-12.exp(290/T) | - | JPL |
| **66** | Cl + CH_4_ (+O_2_)--- HCl + CH_3_O_2_ | k(T) = 7.3E-12.exp(-1280/T) | - | JPL |
| **67** | Cl + C_2_H_6_ --- HCl + EtOO | k(T) = 7.2E-11.exp(-70/T) | - | JPL |
| **68** | Cl + HCHO (+O_2_) --- HCl + HO_2_ + CO | k(T) = 8.1E-11.exp(-30/T) | - | JPL |
| **69** | Cl + CH_3_CHO (+O_2_) --- HCl + CH_3_CO_3_ | k = 8.0E-11 | - | IUPAC |
| **70** | Cl + CH_3_OH (+O_2_) --- HCl + HO_2_ + HCHO | k = 5.5E-11 | - | JPL |
| **71** | Cl + CH_3_OOH --- HCl + HCHO + OH | k = 5.9E-11 | - | IUPAC |
| **72** | ClO + CH_3_O_2_ --- Cl + HCHO + HO_2_ | k = 3.3E-12 | - | JPL |
| **73** | Cl + (CH_3_)_2_S --- products | k = 3.4E-10 | - | IUPAC |
| **74** | ClO + ClO --- Cl + Cl + O_2_ | k(T) = 3.0E-11.exp(-2450/T) | - | JPL |
| **75** | ClO + ClO --- Cl_2_ + O_2_ | k(T) = 1.0E-12.exp(-1590/T) | - | JPL |
| **76** | Cl_2_ + OH --- HOCl + Cl | k(T) = 2.6E-12.exp(-1100/T) | - | JPL |
| **77** | ClO + OH --- Cl + HO_2_ | k(T) = 7.4E-12.exp(270/T) | - | JPL |
| **78** | ClO + BrO --- OClO + Br | k(T) = 1.6E-12.exp(-430/T) | - | IUPAC |
| **79** | ClO + BrO --- BrCl + O_2_ | K(T) = 5.8E-13.exp(-170/T) | - | IUPAC |
|  |  |  |  |  |
|  | **Photolysis** |  |  |  |
| **80** | ClO + hv --- Cl + O(3P) | - | Calculated from abs. cross section | JPL |
| **81** | HOCl + hv --- Cl + OH | - | Calculated from abs. cross section | JPL |
| **82** | ClONO_2_ + hv --- ClO + NO_2_ | - | Calculated from abs. cross section | JPL |
| **83** | ClONO_2_ + hv --- Cl + NO_3_ | - | Calculated from abs. cross section | JPL |
| **84** | ClNO_2_ + hv --- Cl + NO_2_ | - | Calculated from abs. cross section | JPL |
| **85** | Cl_2_ + hv --- Cl + Cl | - | Calculated from abs. cross section | JPL |
| **86** | OClO + hv --- ClO + O(3P) | - | Calculated from abs. cross section |  |
| **87** | BrCl + hv --- Br + Cl | - | Calculated from abs. cross section |  |
|  |  |  |  |  |
|  | **Termolecular** |  |  |  |
| **88** | ClO + NO_2_ --- ClONO_2_ | k_0_(T) = 1.8E-31(T/300)^-3.4^  k_∞_(T) = 1.5E-11(T/300)^-1.9^ | - | JPL |
| **89** | Cl + NO_2_ --- ClNO_2_ | K_0_(T) = 1.8E-31(T/300)^-2^  k_∞_(T) = 1.0E-10(T/300)^-1^ | - | JPL |

**Text S2 : Surface measurements of chlorine VSLS**

A surface mixing ratio boundary condition was imposed in the model for chloroform (CHCl_3_), dichloromethane (CH_2_Cl_2_) and tetrachloroethene (C_2_Cl_4_). For each of these source gases, this boundary condition varied with latitude (5 bands, >60°N, 30-60°N, 0-30°N, 0-30°S, >30°S) and annually based on available surface measurements from two global monitoring networks; the Advanced Global Atmospheric Gases Experiment (AGAGE) and the National Oceanic and Atmospheric Administration's (NOAA’s) Earth System Research Laboratory (ESRL). NOAA/ESRL data is an update to that reported in Montzka et al. [2011].

***Note on other chlorinated VSLS**

Both trichloroethene (C_2_HCl_3_) and 1,2-dichloroethane (CH_2_ClCH_2_Cl) were also considered in a model experiment (EXP3, 2010-2013 only, see main paper). A comprehensive long-term surface record of these species is unavailable and therefore their surface abundance was scaled to give reasonable agreement with observed values in the upper troposphere. We assumed 0.5 ppt of surface C_2_HCl_3_ and 10.0 ppt of surface CH_2_ClCH_2_Cl. Both values fall within previously reported surface ranges given by the WMO Scientific Assessment of Ozone Depletion 2010, based on compiled aircraft data.

**Table S7:** Annual mean surface mixing ratio [ppt] of CHCl_3_ in 5 latitude bands calculated from AGAGE surface observations (<http://agage.eas.gatech.edu/data.htm>).

| **Latitude** | **Year** | | | | | | | | |
| --- | --- | --- | --- | --- | --- | --- | --- | --- | --- |
|  | **2005** | **2006** | **2007** | **2008** | **2009** | **2010** | **2011** | **2012** | **2013** |
| **>60°N** | 12.0 | 12.0 | 12.0 | 12.0 | 12.0 | 12.0 | 11.6 | 9.9 | 11.8 |
| **30-60°N** | 11.0 | 10.8 | 10.5 | 10.4 | 10.2 | 11.0 | 10.7 | 10.8 | 10.8 |
| **0-30°N** | 6.7 | 7.1 | 6.6 | 7.5 | 6.9 | 7.2 | 6.6 | 7.3 | 7.6 |
| **0-30°S** | 5.4 | 5.3 | 5.0 | 4.4 | 3.1 | 2.8 | 4.5 | 4.8 | 5.1 |
| **>30°S** | 5.3 | 5.3 | 5.6 | 5.5 | 5.3 | 5.4 | 5.6 | 5.0 | 5.3 |

**Table S8** Annual mean surface mixing ratio [ppt] of CH_2_Cl_2_ in 5 latitude bands calculated from NOAA/ESRL surface observations (<http://www.esrl.noaa.gov/gmd/dv/ftpdata.html>).

| **Latitude** | **Year** | | | | | | | | |
| --- | --- | --- | --- | --- | --- | --- | --- | --- | --- |
|  | **2005** | **2006** | **2007** | **2008** | **2009** | **2010** | **2011** | **2012** | **2013** |
| **>60°N** | 33.4 | 35.4 | 37.6 | 41.0 | 41.4 | 46.4 | 46.3 | 47.9 | 59.1 |
| **30-60°N** | 35.4 | 37.3 | 40.6 | 42.8 | 42.6 | 48.6 | 48.4 | 50.9 | 62.4 |
| **0-30°N** | 27.1 | 28.4 | 32.6 | 34.3 | 35.3 | 41.0 | 39.9 | 43.5 | 53.0 |
| **0-30°S** | 11.6 | 13.4 | 13.6 | 14.2 | 13.9 | 14.9 | 15.6 | 16.4 | 19.3 |
| **>30°S** | 9.6 | 9.8 | 11.1 | 11.9 | 11.8 | 12.6 | 13.5 | 13.6 | 15.1 |

**Table S9:** Annual mean surface mixing ratio [ppt] of C_2_Cl_4_ in 5 latitude bands calculated from NOAA/ESRL surface observations (<http://www.esrl.noaa.gov/gmd/dv/ftpdata.html>).

| **Latitude** | **Year** | | | | | | | | |
| --- | --- | --- | --- | --- | --- | --- | --- | --- | --- |
|  | **2005** | **2006** | **2007** | **2008** | **2009** | **2010** | **2011** | **2012** | **2013** |
| **>60°N** | 3.7 | 3.6 | 3.3 | 3.5 | 2.8 | 2.9 | 2.5 | 2.3 | 2.3 |
| **30-60°N** | 5.5 | 5.4 | 4.9 | 4.5 | 4.1 | 4.1 | 3.6 | 3.2 | 3.6 |
| **0-30°N** | 2.2 | 2.2 | 2.1 | 2.0 | 1.9 | 1.9 | 1.6 | 1.5 | 1.6 |
| **0-30°S** | 0.6 | 0.7 | 0.6 | 0.8 | 0.7 | 0.6 | 0.5 | 0.5 | 0.6 |
| **>30°S** | 0.5 | 0.5 | 0.5 | 0.6 | 0.5 | 0.5 | 0.4 | 0.4 | 0.4 |

**Figure S3:** Simulated annual and zonal mean latitude-pressure cross section of formyl chloride (CHClO) mixing ratio (ppt) in 2013.

**Supporting References**

Atkinson, R., et al., (2008), Evaluated kinetic and photochemical data for atmospheric chemistry: Volume iv. gas phase reactions of organic halogen species, *Atmos. Chem. Phys*., 8, 4141–4496, doi:10.5194/acp-8-4141-2008.

Biggs, P., C. E. Canosa-Mas, C. J. Percival, D. E. Shallcross, and R. P. Wayne (1999), A study of the self reaction of CH_2_ClO_2_ and CHCl_2_O_2_ radicals at 298 k, *Int. J. Chem. Kinet*., 31(6), 433–444, doi:10.1002/(SICI)1097-4601(1999)31:6<433::AID-KIN5>3.0.CO;2-E

Brudnik, K., D. Wjcik-Pastuszka, J. Jodkowski, and J. Leszczynski (2008), Theoretical study of the kinetics and mechanism of the decomposition of trifluoromethanol, trichloromethanol, and tribromomethanol in the gas phase, *J. Mol. Model*., 14(12), 1159–1172, doi:10.1007/s00894-008-0358-0.

Catoire, V., R. Lesclaux, W. Schneider, and T. Wallington (1996), Kinetics and mechanisms of the self-reactions of CCl_3_O2 and CHCl_2_O_2_ radicals and their reactions with HO_2_, *J. Phys. Chem*., 100(34), 14,356–14,371, doi:10.1021/jp960572z.

Hou, H., L. Deng, J. Li, and B. Wang, A systematic computational study of the reactions of HO_2_ with RO_2_: the HO_2_ + CH_2_ClO_2_, CHCl_2_O_2_, and CCl_3_O_2_ reactions, The Journal of Physical Chemistry A, 109(41), 9299–9309, doi:10.1021/jp052718c.

Jowko, A., K. Wnorowski, J. Kowalczyk, and K. Wojciechowski, The kinetics of the radical reactions in gaseous chlorofluorohydrocarbons, *Radiat. Phys. Chem*., 67(3-4), 549–554, doi:10.1016/S0969-806X(03)00104-X, 2003.

Kindler, T. P., W. L. Chameides, P. H. Wine, D. M. Cunnold, F. N. Alyea and J. A. Franklin (1995), The fate of atmospheric phosgene and the stratospheric chlorine loadings of its parent compounds: CCl_4_, C_2_Cl_4_, C_2_HCl_3_, CH_3_CCl_3_, and CHCl_3_, *J. Geophys. Res. [Atmos.]*, 100(D1), 1235-1251, doi:10.1029/94JD02518.

Ko, K., and W. Poulet et al. (2003), Halogenated very short-lived substances, in: Scientific Assessment of Ozone Depletion: 2002, Global Ozone Research and Monitoring Project, Report No. 50, Chapt. 2, World Meteorological Organization, Geneva.

Krysztofiak, G., V. Catoire, G. Poulet, V. Marcal, M. Pirre, F. Louis, S. Canneaux, and B. Josse (2012), Detailed modeling of the atmospheric degradation mechanism of very-short lived brominated species, *Atmos. Env*., 59(0), 514–532, doi:http://dx.doi.org/10.1016/j.atmosenv.2012.05.026.

Montzka, S. A., M. Krol, E. Dlugokencky, B. Hall, P. Jöckel, and J. Lelieveld (2011),

Small interannual variability of global atmospheric hydroxyl, *Science*, 331, 67–69, doi:

10.1126/science.1197640.

# Orkin, V. L., V. G., Khamaganov, S. N., Kozlov, and M. J. Kurylo (2013), Measurements of rate constants for the OH reactions with bromoform (CHBr_3_), CHBr_2_Cl, CHBrCl_2_, and epichlorohydrin (C_3_H_5_ClO), *J. Phys. Chem. A*., 117(18), 3809-18, doi: 10.1021/jp3128753.

Sander, S., et al., (2011), Chemical Kinetics and Photochemical Data for Use in Atmospheric Studies, Evaluation Number 17, JPL Publication 10-6, Jet Propulsion Laboratory.

Simonaitis, R., and J. Heicklen (1979), The reactions of CCl_3_O_2_ with NO and NO_2_ and the thermal decomposition of CCl_3_O_2_NO_2_, *Chem. Phys. Lett*., 62(3), 473–478, doi: ttp://dx.doi.org/10.1016/0009-2614(79)80744-7.

Tuazon, E.C., R. Atkinson, S.M. Aschmann, M.A. Goodman, and A.M. Winer (1988), Atmospheric reactions of chloroethenes with the OH radical, Int. J. Chem. Kinet., 20, 241-265, 1988.
